# In-situ observation of collective bubble collapse dynamics in a quasi-two-dimensional foam

**DOI:** 10.1038/s41598-019-41486-6

**Published:** 2019-03-26

**Authors:** Naoya Yanagisawa, Rei Kurita

**Affiliations:** Department of Physics, Tokyo Metropolitan University, 1-1 Minamioosawa, Hachiouji-shi, Tokyo, 192-0397 Japan

## Abstract

The stability of foams is an important subject not only for fundamental science, but for applications in daily life. The most destructive phenomenon underpinning foam collapse is a collective bubble collapse, yet the mechanism behind this is unclear. In this study, we clarify the dynamics of the collective bubble collapse in a quasi-two-dimensional foam by *in*-*situ* observation with a high speed camera. We find two modes for collective bubble collapse: one is the propagation of liquid film breakage via impact with the stream of another broken liquid film. The other is breakage of a distant liquid film due to penetration by a liquid droplet, emitted by impact with the flow of a broken liquid film. As the liquid fraction increases, the velocity of liquid droplets decreases. Instead of penetration, the liquid droplet bounces like a billiard ball or it is absorbed into other films.

## Introduction

A liquid-gas foam is a non-equilibrium jammed state system composed of gas bubbles and liquid films. Foam, which has a cell structure of gas bubbles, is widely used in daily life e.g. in food and cosmetic applications (ice-cream, food mousse and whipped cream, shaving and styling foams), pharmaceutical products (topical and rectal foams), bio-technologies (foam in bio-fermenters), chemical technology (froth flotation) amongst many other applications^1–13^. Therefore, it is a matter of importance for a variety of fields to understand and control foam stability.

The foam is a metastable non-equilibrium state and eventually collapses as time elapses^5,12^. The dynamics of foams is governed by three different processes: these are coarsening, drainage, and collapse in order of evolution rate. Coarsening, where the number of bubbles in the foam decreases and the average bubble size grows, occurs due to different pressures between the bubbles. Drainage leads to thinning of the liquid films due to gravity. Drainage and coarsening are well studied and understood from both experimental and theoretical perspectives. Bubble collapse has also been investigated^9,14–16^. A previous study showed that bubble collapse occurred when a lower coalescence limit was reached in liquid volume fraction^9^. A relationship between bubble collapse and T1 rearrangement has also been studied by injecting air into a system^14^. In addition, when bubbles burst in the foam, a crackling sound is emitted. By measuring and analyzing the acoustic emissions from foams, it has been suggested that a collective bubble collapse (CBC) phenomenon occurs during the collapse of foams^5,12^. This is sometimes called “cascade collapse”. A similar effect has also been observed in emulsions which are even more difficult to analyze due to smaller droplet size. Although CBC plays a role in foam stability, the mechanism behind CBC is still unclear; a framework and perspective for understanding CBC is yet to be established.

In this article, we investigate the mechanism behind CBC. Having prepared a quasi-two dimensional foam, a single bubble is popped at the edge; details are given in the Materials and Methods section. We then directly observe the CBC phenomenon by using a high speed camera. We identify two mechanisms behind the propagation of a rupture event; “propagating” and “penetrating” modes. As $\varphi$ increases, it becomes harder for liquid droplets to penetrate liquid films, leading to droplets bouncing off films due to their elasticity, or being absorbed into the film.

## Results

**Figure 1:**
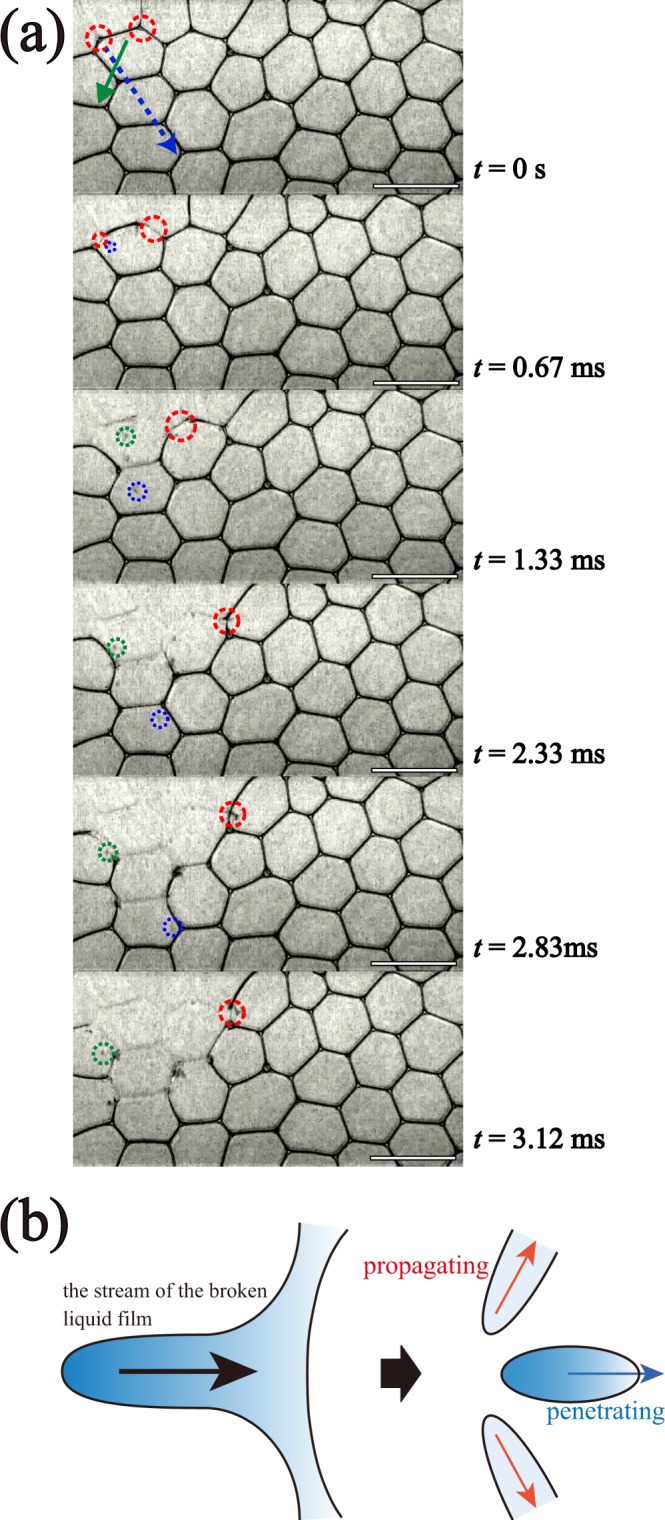
(**a**) Enlarged successive images of the CBC process for $\varphi$ = 0.0099 until *t* = 3.12 ms. The white bar corresponds to 5 mm. Red dashed circles show the breaking of the liquid film by the propagating mode. Blue and green dashed circles show liquid droplets emitted by the impact of the stream of the broken liquid. The arrows show trajectories of those liquid droplets. (**b**) Schematic image for CBC. The impact of the stream of the broken liquid induces the breaking of the liquid film (propagating mode) and the emission of a liquid droplet (penetrating mode).

Firstly, we perform *in*-*situ* observation of the CBC process for different $\varphi$. Figure 1(a) shows enlarged successive images of the CBC process from time *t* = 0 ms to 3.12 ms for $\varphi$ = 0.0099 [see Supplementary Movie 1]. We puncture a bubble out side the foam using a capillary glass needle with a very small amount of silicon grease on the tip. Collapse of the bubbles is firstly observed at an outer edges since rapture of the foam film proceeds on the outer edge. It can be called a surface effect. Then we find two modes for CBC collapse in bulk: one is a ‘propagating’ mode, the other is a ‘penetrating’ mode. When a liquid film breaks, the broken liquid film is rapidly absorbed by the Plateau border. Due to the strong absorbing impact, another liquid film sharing the same Plateau border is also broken, indicated by the red circle in Fig. 1(a). This is the propagating mode of liquid film breakage [see Fig. 1(b)]. At the same time, when the broken liquid film is absorbed by the Plateau border, a liquid droplet is emitted, indicated by the blue and green circles in Fig. 1(a). The droplets run into the inside of the foam as shown by the arrows in Fig. 1(a), and penetrate a distant liquid film. The velocity of the liquid droplets *V*_*d*_ is found to be approximately 3 m/s. This penetration impact induces breaking the distant liquid film. This is the penetrating mode [see Fig. 1(b)]. The broken liquid film induces another broken film by both propagating and penetrating modes. The CBC collapse occurs as a result of the repeated breakage of liquid films via the two modes.

**Figure 2:**
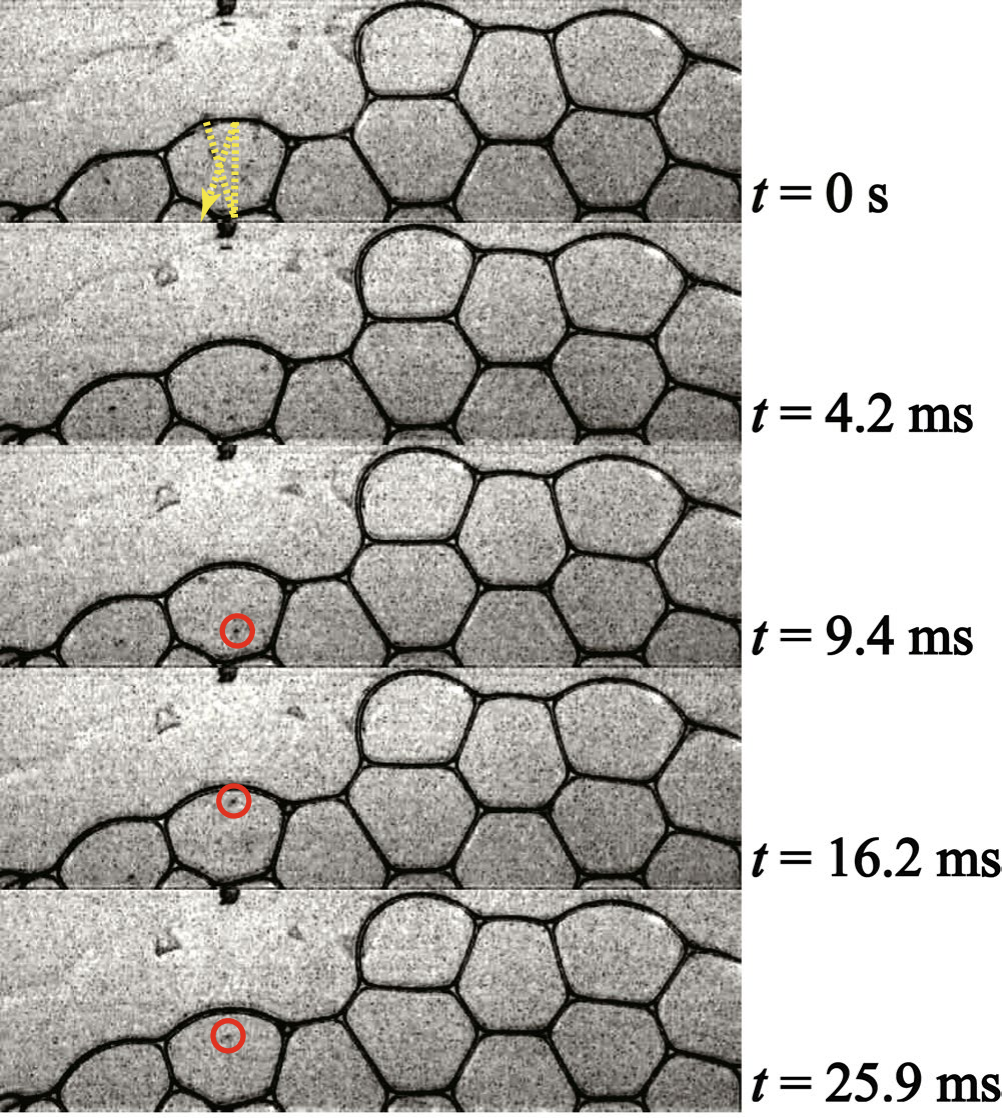
Enlarged successive images of the bouncing process for $\varphi$ = 0.015 until *t* = 25.9 ms. The white bar corresponds to 5 mm. Red circles show the liquid droplet emitted by the propagating mode. Yellow dashed line shows the trajectory of the liquid droplet.

**Figure 3:**
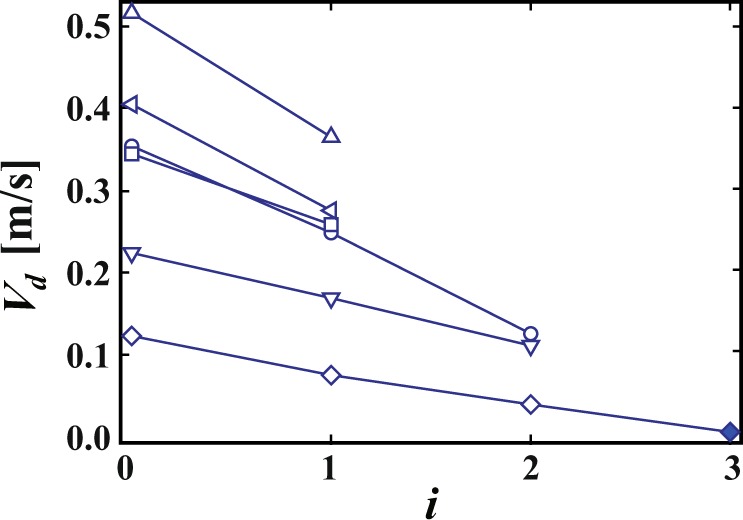
The velocity of the liquid droplet *V*_*d*_ as a function of a number of collisions with the liquid film *i*. *V*_*d*_ decreases after each impact. The coefficient of restitution of the liquid film is 0.50~0.74. The droplet is finally absorbed into the liquid film, indicated by the filled symbol.

As $\varphi$ increases ($\varphi$ ≥ 0.015), the probability of a liquid droplet being emitted decreases. The velocity of liquid droplets also decreases; it becomes harder for liquid droplets to penetrate distant liquid films. Instead of penetration, the liquid droplet bounces like a billiard ball [see Supplementary Movie 2]. Figure 2 shows how the liquid droplet bounces over 30 ms; the dashed line shown in the top indicates the trajectory it follows. Measuring *V*_*d*_ after each bounce, we can plot *V*_*d*_ as a function of the number of impacts *n*_*i*_, as shown in Fig. 3. We find that *V*_*d*_ decreases with each impact. We define the coefficient of restitution of the liquid film as $e=|{V}_{d}(i+1)|/|{V}_{d}(i)|$ where $|{V}_{d}(i)|$ is a droplet velocity after the *i*-th bounce and we obtain that *e* = 0.50~0.74. After the droplet bounces several times, it is absorbed into the liquid film, as indicated by the filled symbol in Fig. 3. We note that a bouncing and absorption of the drop is similar to results in the soap film^17^.

As $\varphi$ is increased further ($\varphi$ > 0.022), the liquid film is successfully absorbed into the Plateau border, and liquid droplets are not emitted. The bubbles at the outer edges of the foam are broken by the surface effect even with greater $\varphi$; however, fewer bubbles are broken, and the collapse stops quickly. As a result, a CBC does not occur. This suggests that the penetrating mode is crucial for CBC occurrence.

**Figure 4:**
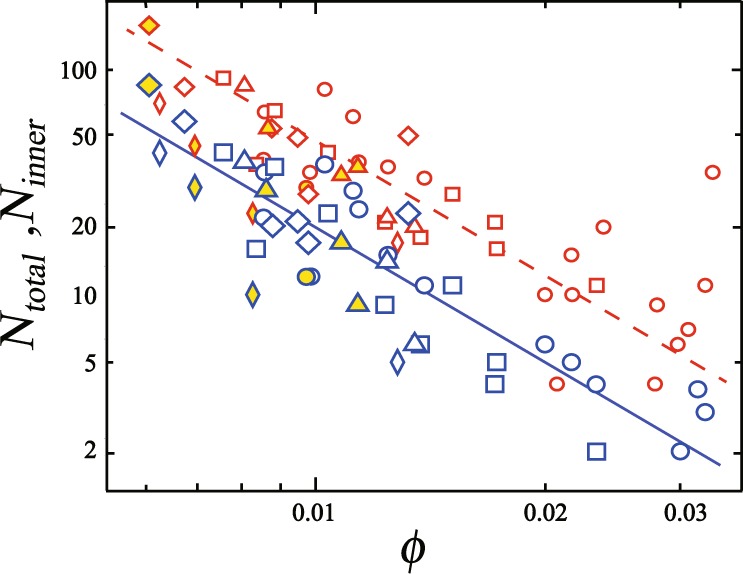
$\varphi$ dependence of *N*_*total*_ (red symbols) and *N*_*inner*_ (blue symbols). *N*_*total*_ and *N*_*inner*_ decreases with ${\varphi }^{-\gamma }$; fit lines reveal that *γ* is 2.3 ± 0.36. Triangles, circles, and squares correspond to *N*_*total*_ and *N*_*inner*_ for glycerol concentrations of 9.4 wt%, 17.8 wt% and 29 wt%, respectively. Thin diamond symbols correspond to *N*_*total*_ and *N*_*inner*_ for a circular foam, with a glycerol concentration of 17.8 wt%. Diamond symbols correspond to *N*_*total*_ and *N*_*inner*_ for a large foam. The filled symbols correspond to *N*_*total*_ and *N*_*inner*_ when the CBC phenomenon occurs spontaneously before a bubble is popped with a needle. This means that the CBC phenomenon is independent of the use of silicone grease.

Next, we investigate the $\varphi$ dependence of the number of collapsing bubbles quantitatively. A number of total collapsing bubbles *N*_*total*_ includes the bubble at the outer edge of the foam, which are collapsed by the surface effect, not only the propagation mode and the penetration mode in bulk. Since we focus on the CBC in bulk, we count a number of the collapsing bubbles inside the foam *N*_*inner*_, which excludes the surface effect. Thus, *N*_*inner*_ corresponding to the number of bubbles broken by both propagating mode and penetrating mode in bulk. We count the number of broken bubbles, from the first to last bubble broken; this occurs over approximately 0.04 seconds. This number includes double collapsing events^18^; the time scale of the CBC in our experiment is consistent with that reported in ref.^18^. Figure 4 shows *N*_*total*_ (red) and *N*_*inner*_ (blue) as a function of $\varphi$. Triangles, circles, and squares correspond to *N*_*total*_ or *N*_*inner*_ at *N*_*f*_ ~ 200 for glycerol concentrations of 9.4 wt%, 17.8 wt% and 29 wt%, respectively. *N*_*f*_ is the number of bubbles in the whole foam. As shown, both *N*_*total*_ and *N*_*inner*_ decreases as $\varphi$ increases. From the power law fit, we find that ${N}_{inner}\propto {\varphi }^{-{\gamma }_{e}}$ with *γ*_*e*_ = 2.3 ± 0.36. Note that *N*_*inner*_ is quite small for $\varphi$ > 0.015; CBC collapse seems to occur in dry states, where the boundary between dry and wet foams is $\varphi \simeq 0.05$ in a quasi-two-dimensional foam^13,19^. We also find that both *N*_*total*_ and *N*_*inner*_ is independent of the glycerol concentration below 29 wt%, that is, with a change in viscosity. This suggests that the behavior of the liquid film is primarily inertial rather than viscous. It becomes difficult to puncture a bubble when the glycerol concentration is more than 40 wt% and CBC is not observed. We also note that *N*_*total*_ (or *N*_*inner*_) collapses into the power law curve although the size distribution of the bubbles varies in each experiment. We also investigated the number of collapsing bubbles with a larger foam (*N*_*f*_ ~ 500), as shown by the diamond symbols in Fig. 4. This suggests that *N*_*total*_ and *N*_*inner*_ are independent of *N*_*f*_. We also considered the fact that we used silicone grease in order to induce the first bubble breakage; the grease may also affect the collapse of the other bubbles in the CBC process^20–22^. Thus, we measured *N*_*total*_ and *N*_*inner*_ without using silicone grease, that is, *N*_*total*_ and *N*_*inner*_ when the CBC phenomenon spontaneously occurs. This is shown by the filled symbols in Fig. 4. We find that the number of collapsing bubbles without using silicone grease collapses onto the existing curve, suggesting that the CBC phenomenon is independent of the use of the silicone grease.

**Figure 5:**
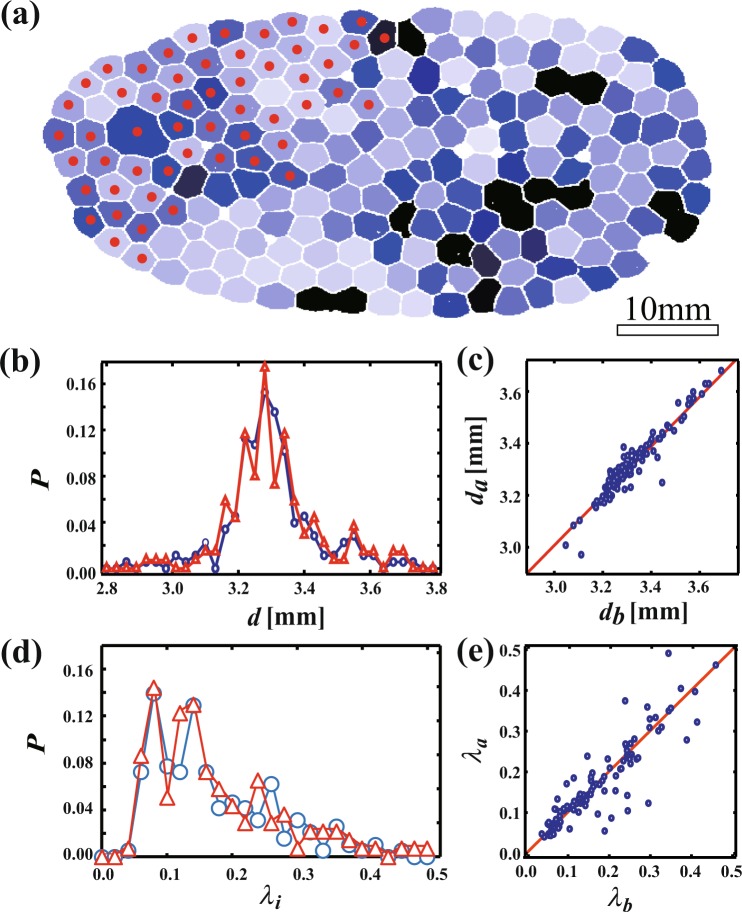
(**a**) Relationship between bubbles broken during CBC and deformed bubbles in the foam, at $\varphi$ = 0.0086. The bubble color is darker for larger deformations, lighter for smaller deformations. Red dots indicate bubbles broken during the CBC. (**b**) Probability of a diameter *d* before (blue line) and after CBC (red line). (**c**) A relation between bubble diameters before CBC *d*_*b*_ and after CBC *d*_*a*_. It means that CBC is independent of the size distribution. (**d**) Probability of *λ*_*i*_ before CBC *λ*_*b*_ and after CBC *λ*_*a*_. (**e**) A relation between *λ*_*i*_ before and after CBC. It is found that the shape is not related with the CBC process.

When $\varphi$ is small, it is likely that the shape of each bubble is anisotropic and that the distorted bubbles reconnected in a chain-like form^13^. Bubbles with larger size than a mean size or anisotropic shape have large excess surface energy and one might think that such larger or anisotropic bubbles tend to collapse easily. Thus, we investigate the relationship between CBC and the size distribution and between CBC and the shapes of the bubbles. We use a parameter *λ*_*i*_ to characterize the anisotropy of bubble *i*^13^. *λ*_*i*_ is defined as

1 $${\lambda }_{i}=\sqrt{\frac{1}{n}{{\rm{\Sigma }}}_{j=1}^{n}{({r}_{j}-\bar{r})}^{2}},$$

where *j* is a pixel at the edge of the bubble, *n* is a total number of the pixel *j*, *r*_*j*_ is a distance between the center of bubble *i* and the pixel *j* and $\bar{r}$ is a mean distance of *r*_*j*_. *λ*_*i*_ = 0 when the bubble *i* is circular, whereas *λ*_*i*_ > 0 if the bubble is anisotropic. Figure 5(a) is a foam with $\varphi$ = 0.0086 before the CBC. The bubble color is set to black when *λ*_*i*_ is larger, and closer to white when *λ*_*i*_ is smaller. Red dots indicate the bubbles collapsed during the CBC event. We find that the bubbles located at the left of the foam are uniformly collapsed. Figure 5(b) shows a probability distribution as a function of a mean bubble diameter of a bubble *i d*_*i*_ before and after the CBC for the bubbles. *d*_*i*_ is computed by averaging a distance between a center and an interface. The shape of the probability distribution after the CBC is the same as that before the CBC. Figure 5(c) shows that *d*_*i*_ after CBC *d*_*a*_ as a function of *d*_*i*_ before CBC *d*_*b*_. It is found that *d*_*a*_ = *d*_*b*_ as shown a line in Fig. 5(c) and it means that each bubble size is unchanged during CBC. We also shows the probability of *λ*_*i*_ before and after the CBCs in Fig. 5(d). As shown in Fig. 5(d), the shape of the probability distribution after the CBC is also the same as that before the CBC. We compute *λ*_*i*_ before CBC *λ*_*b*_ and after CBC *λ*_*a*_ and we find that ${\lambda }_{a}={\lambda }_{b}$ for almost all bubbles shown as in Fig. 5(e). It means that the shape of the bubble is not relaxed after the collapsing occurs and it is consistent with a previous report^13^. This suggests that the bubbles collapsed during the CBC are also independent of the shape of the bubbles. In addition, we investigate the $\varphi$ dependence of *N* when the shape of the whole foam is circular. These are shown by the thin diamond symbols in Fig. 4. According to a previous report, the shape of each bubble corresponds to the shape of the whole foam^13^. Thus, this result supports the hypothesis that the CBC phenomenon is independent of the size distribution and the shape of each bubble. It is also reported in ref.^18^ that the collapsing bubbles along the air/foam interfaces have no particular topological selection i.e. the bubbles popped are independent of bubble shape. This is consistent with the propagating and penetrating modes being induced by the mechanical impact of the broken liquid film rather than excess surface energy due to the larger and anisotropic shape. Here we should note that the mean bubble size should affect the CBC process. We will discuss it later.

**Figure 6:**
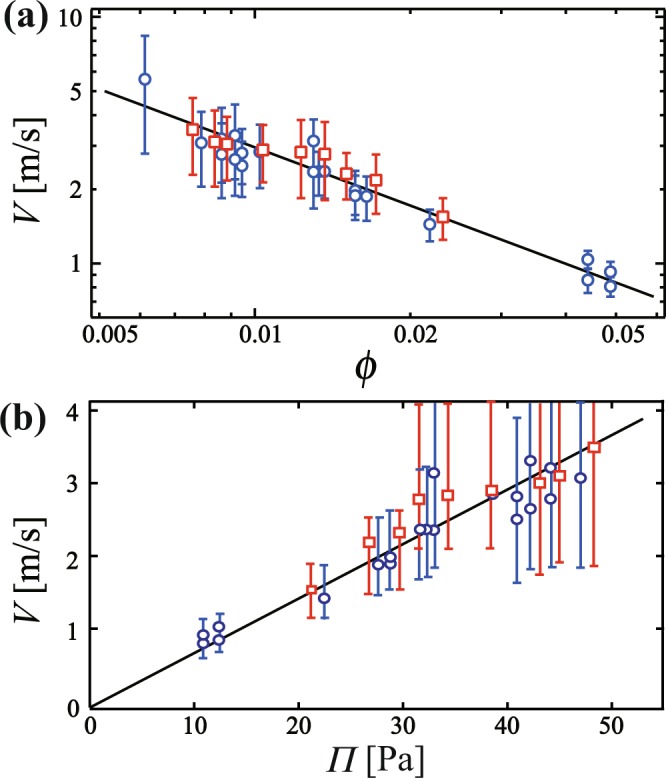
(**a**) Stream velocity *V* as a function of $\varphi$, shown as a log-log plot. We find that *V* is proportional to ${\varphi }^{-0.76}$. Circles and squares correspond to *V* for glycerol concentrations of 17.8 wt% and 29 wt%, respectively. (**b**) *V* as a function of osmotic pressure ${\rm{\Pi }}$. ${\rm{\Pi }}$ is calculated by Eq. (2). It is found that *V* is proportional to ${\rm{\Pi }}$.

In order to evaluate the mechanical impact of the broken liquid film, we measure the velocity of the broken liquid film *V* in the foam with 17.8 wt% glycerol when it is absorbed. We define *V* as *l*/*t*, where *l* is the length of the liquid film and *t* is the time it takes for the liquid film to be absorbed, from start to finish. Figure 6(a) shows the $\varphi$ dependence of *V* as a log-log plot. We find that *V* is approximately 1~10 m/s. The order of *V* is consistent with that in inertial liquid films; note that the velocity in viscous liquid films is ~mm/s^23,24^. From the fit, we obtain *V* = *V*_0_ × $\varphi$^−*α*^, where *V*_0_ = 0.083 ± 0.018 m/s and *α* = 0.76 ± 0.07. We also obtain *V* in a foam with 29 wt% glycerol, as shown by the square symbols in Fig. 6. We found that *V* with 29 wt% glycerol is almost the same as *V* with 17.8 wt% glycerol. This result suggests that the behavior of the liquid film is inertial rather than viscous. The rapid decrease of *V* as a function of $\varphi$ is consistent with our observations as shown in Fig. 1. With increasing $\varphi$, the emitted droplet is bounced or absorbed due to decreasing of *V*. Here we compare the Taylor-Culick velocity *V*_*TC*_. *V*_*TC*_ can be described as ${V}_{TC}=\sqrt{2\sigma /\rho h}$ where *σ* and $\rho$ are the surface tension and the density of the liquid, respectively, and *h* is the thickness of the film^25,26^. *α* should be 0.25 if *V* is the Taylor-Culick velocity^25,26^ since a local liquid film thickness *h* is proportional to ${\varphi }^{0.5}$^27^. It is not consistent with our results (*α* = 0.76). We also investigate the relation between the velocity and osmotic pressure ${\rm{\Pi }}$. The relationship between ${\rm{\Pi }}$ and $\varphi$ has been studied previously^28–30^. According to ref.^28^, ${\rm{\Pi }}$ for 2 dimensional foam becomes

2 $${\rm{\Pi }}=\frac{\sigma }{R}{(\frac{1-\varphi }{1-{\varphi }_{J}})}^{0.5}[{(\frac{{\varphi }_{J}}{\varphi })}^{0.5}-1],$$

where *σ* is a surface tension and *R* is a mean radius of the bubble and ${\varphi }_{J}$ is a jamming point and ${\varphi }_{J}$ = 0.16 in 2 dimension. In our experiment, we used *σ* = 37 mN/m and *R* = 1.7 mm. In the Hele-Shaw cell, a thin liquid layer exists on the wall. Thus we should subtract a fraction of the wetting layer from the liquid fraction in order to use the equation. Then we estimate ${\rm{\Pi }}$ with assuming that a thickness of the thin wetting layer is 1 *μ*m. Figure 6(b) shows *V* as a function of ${\rm{\Pi }}$; it is found that *V* is proportional to ${\rm{\Pi }}$. We confirm the linear relation between *V* and ${\rm{\Pi }}$ even though we estimate ${\rm{\Pi }}$ with changing the thickness of the wetting layer from 0 *μ*m to 2 *μ*m. From the linear relation, we consider that the driving force of the absorption is negative pressure in the foam film. Furthermore, we also compute $\varphi$ dependence of ${\rm{\Pi }}$ as shown in Fig. S1 in the Supplementary. We find that ${\rm{\Pi }}\sim {\varphi }^{-0.75}$ for 0.005 < $\varphi$ < 0.05. Then we obtain $V\sim {\rm{\Pi }}\sim {\varphi }^{-0.75}$ and it is consistent with our observation.

**Figure 7:**
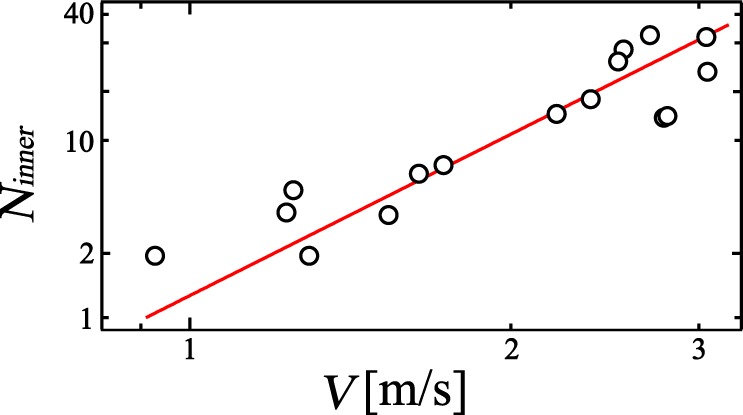
*N*_*inner*_ dependence of *V*, shown as a log-log plot. We find *N*_*inner*_ = 1.54 (*V*/*V*_1_)^2.9^ where we set *V*_1_ = 1 m/s.

Furthermore, we also look at the *V* dependence of *N*_*inner*_ in Fig. 7. We find that *N*_*inner*_ drastically increases with increasing *V*. This means that CBC is strongly correlated with the stream velocity of the liquid film. Again, it is consistent with the notion that CBC is induced by the mechanical impact of the liquid film. We also obtain *N*_*inner*_ = *N*_0_ × (*V*/*V*_1_)^*β*^ where we set *V*_1_ = 1 m/s. The variable parameters are found from the fit, *N*_0_ = 1.54 ± 0.56 and *β* = 2.9 ± 0.40. *N*_*inner*_ steeply increases with increasing *V*. It supports that the penetration mode is crucial for the CBC. From the relation $\gamma =\alpha \beta$, we compute the power law relationship ${N}_{inner}\propto {\varphi }^{-{\gamma }_{c}}$ where *γ*_*c*_ = 2.2 ± 0.50. The calculated *γ*_*c*_ is consistent with *γ*_*e*_ (2.3); this gives us more assurance as to the reliability of these experimental results.

## Discussion

Here, we discuss the linear relationship between *V* and ${\rm{\Pi }}$. We start from the Navier-Stokes equation for the liquid film. We set *x* axis as a direction of absorption and we neglect velocity in a perpendicular direction to absorption.

3 $$\frac{\partial V}{\partial t}+V\frac{\partial V}{\partial x}=-\,\frac{1}{\rho }\nabla P+\nu {\nabla }^{2}V-\frac{kV}{\rho },$$

where *P* is a pressure and $P=-\,{\rm{\Pi }}$, *ν* is a kinematic viscosity, and *k* is a coefficient. −*kV* is a term for resistance force between a glass substrate and the liquid film. From our observations, *V* seems almost constant with time. We cannot observe a rim during an absorption, which differs from an absorption of a soap film (Taylor-Culick case). It suggests that local volume is unchanged and then *V* is almost homogeneous in *x* direction in the liquid film. Then we also obtain that ${\nabla }^{2}V=0$ and it is consistent with our observation, which *V* is independent of *ν*. Then we obtain that

4 $$\nabla {\rm{\Pi }}=kV.$$

Then $\nabla {\rm{\Pi }}\sim {\rm{\Pi }}/l$, where *l* is a length of the liquid film. Thus, we find that

5 $$V\propto {\rm{\Pi }}.$$

Our observation of $V\propto {\rm{\Pi }}$ can be explained by a balance between the negative pressure and the resistance between the liquid film and the glass substrate. It is similar that a velocity of a falling liquid droplet is determined by a balance between a gravity force and air resistance. Thus we consider that *V* is determined by the osmotic pressure, rather than $\varphi$. We note that ${\rm{\Pi }}$ is related with the bubble size *R* according to Eq. (2). Thus *V* depends on *R* and then the number of the collapsing bubbles should be depend on *R*. In near future, we investigate the CBC with changing the mean bubble size, not only the bubble size distribution.

Here we compare other experiments, which the drop penetrates the film without breaking. We introduce the weber number We = $\rho R{V}^{2}/\sigma$, where $\rho$ is density. According to previous reports, the drop penetrates the film without breaking when We > 20^31,32^. We calculate We in the penetration mode of the foam using $\rho$ = 1000 kg/m^3^, *R* = 100 *μ*m, *V* = 3 m/s, and *σ* = 37 mN/m. It is found that We ~ 30 and it is above the critical We. it seems inconsistent with the experiment with the soap film. The liquid film in the foam is quite thin (~100 nm) due to the negative osmotic pressure due to packing in the dry foam^28,29^, while the soap film has several *μ*m^31,32^. When the emitted droplet impacts the liquid film in the foam, the liquid film should be distorted. We consider two possibilities. One is that the liquid film is broken due to thinning by the distortion. The other is that the bending modulus is large due to resistance for thinning. Then, a large hole is created by an emitted droplet, while the soap film is easily bended. Thus we consider that the rapid droplet can break the liquid film in the foam. We should investigate how the droplet penetrates and the liquid film is broken in future.

Next, we discuss the other exponents, *β* and *γ*. To establish a theoretical approach, we consider the probability of a breakage being induced by one penetrating droplet. Since this depends on the droplet velocity and the local liquid fraction, this probability can be expressed as $P(V,\varphi (\overrightarrow{r}))$. Meanwhile, *N*_*inner*_ is not the number of bubbles broken in the first collapse process but a summation over the entire process. Also note that the absorption of the broken liquid film should change the local liquid fraction, since the diffusion of the liquid in the foam is much slower than the CBC dynamics. Thus, we find that *N*_*inner*_ is not a suitable parameter for a theoretical approach and we cannot relate $P(V,\varphi (\overrightarrow{r}))$ to *N*_*inner*_. To reveal the origin of the exponents *β* and *γ*, we may need to investigate the first breakage process distinctly, separated from secondary and higher order processes to obtain an expression for $P(V,\varphi (\overrightarrow{r}))$.

## Summary

To summarize, we perform *in*-*situ* observation of collective bubble collapse in a quasi-two-dimensional liquid-gas foam by using a high speed camera. It was found that there were two crucial modes for the CBC. One is the propagating mode, the liquid-film breaking due to the impact of absorption. The other mode is the penetrating mode, when liquid droplets are emitted due to the impact of absorption and penetrate through other, distant liquid films. We also investigate the CBC phenomenon for different liquid fractions. As the liquid fraction increases, the velocity of the liquid droplet decreases, making it harder to penetrate liquid films. Instead of penetration, the liquid droplets bounce due to the elasticity of the liquid film. When the velocity becomes slower, the liquid droplet is absorbed into the other liquid film. We find that the absorption velocity is proportional to the osmotic pressure; an explanation for the scaling of other parameters is left for future work, along with an examination of the local dynamics of the first breakage to further clarify the collective collapse process.

## Materials and Methods

We used a 14 wt% solution of TTAB (tetradecyl trimethyl ammonium bromide) in glycerol and deionized water. In this article, we mainly show the results of foams with 17 wt% glycerol. The foams are created by using a capillary glass tube equipped with an air pump. We put the foam at the center of a glass plate and cover it with another glass plate. The sample thickness is controlled to 2.2 mm by using a spacer. This sample cell is horizontal; the foam is regarded as quasi two dimensional. The diameter of the bubbles is about 3~5 mm, and the number of the bubbles *N*_*f*_ is about 150~200 or approximately 500. We punctured a bubble outside the foam using a capillary glass needle with a very small amount of silicon grease on the tip to induce CBC collapse^20–22^. We took images during the collapsing process of the bubbles using a high speed camera (KEYENCE, VW-9000) with a frame rate between 4000 and 10000 fps. The temperature was set to around 20 °C using an air conditioner. Here, we determine the liquid fraction $\varphi$ in quasi-two-dimensional foams by the method described in ref.^19^. It takes only a few minutes to perform the experiment, thus evaporation of the liquid is negligible. We also neglect drainage and coarsening.

## Supplementary information

Supplementary information

Movie for CBC process

Movie for bouncing process
