# Supplementary material for: In-situ observation of collective bubble collapse dynamics in a quasi-two-dimensional foam
Source: Sci Rep. 2019 Mar 26;9:5152. doi: 10.1038/s41598-019-41486-6 (PMC6435646; doi:10.1038/s41598-019-41486-6)
Supplement: Supplementary file 1 — Supplementary information [file 41598_2019_41486_MOESM1_ESM.pdf]

Supplementary Information for

"In-situ observation of collective bubble collapse dynamics in a quasi-two-dimensional foam"

Naoya Yanagisawa and Rei Kurita

Department of Physics, Tokyo Metropolitan University, Tokyo 192-0397, Japan

(Supplementary movie 1) The enlarged movie of the collective bubble collapsing process from time $t = 0$ s to $t = 0.02$ s for $\phi = 0.0099$.

We took this movie by using a high speed camera with the frame rate 6000 fps. This movie is same as figures 1(a) in the main manuscript.

(Supplementary movie 2) The enlarged movie of the bouncing process from time $t = 0$ s to $t = 0.024$ s for $\phi = 0.015$.

We took this movie by using a high speed camera with the frame rate 10000 fps. The red dashed circle in this movie indicates that a liquid droplet bounces like a billiard ball.

Osmotic pressure as a function of ϕ.

The osmotic pressure is computed using Eq. (2) in the main manuscript. A red line corresponds to $\phi^{-0.75}$.

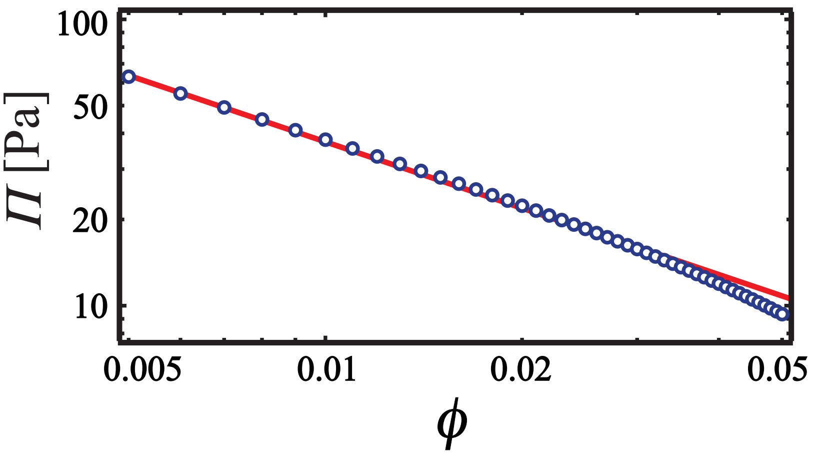

Fig. S1 Osmotic pressure as a function of ϕ. From a power-law fitting, we find that $\Pi \sim \phi^{-0.75}$.
